# 1,5‐Diazacyclooctanes, as Exclusive Oxidative Polyamine Metabolites, Inhibit Amyloid‐*β*(1‐40) Fibrillization

**DOI:** 10.1002/advs.201600082

**Published:** 2016-06-01

**Authors:** Ayumi Tsutsui, Tamotsu Zako, Tong Bu, Yoshiki Yamaguchi, Mizuo Maeda, Katsunori Tanaka

**Affiliations:** ^1^ Biofunctional Synthetic Chemistry LaboratoryRIKENHirosawa, Wako‐shiSaitama351‐0198Japan; ^2^ Bioengineering LaboratoryRIKENHirosawa, Wako‐shiSaitama351‐0198Japan; ^3^ Systems Glycobiology Research GroupRIKEN‐Max Plank Joint Research Center for Systems Chemical BiologyRIKEN Global Research ClusterRIKENHirosawa, Wako‐shiSaitama351‐0198Japan; ^4^ Biofunctional Chemistry LaboratoryA. Butlerov Institute of ChemistryKazan Federal University18 Kremlyovskaya StreetKazan420008Russia; ^5^ JST‐PRESTOHirosawa, Wako‐shiSaitama351‐0198Japan

**Keywords:** acrolein, amyloid, cycloaddition, oxidative stress, polyamine

## Abstract

**Biologically relevant 1,5‐diazacyclooctanes** derived from polyamines and acrolein, inhibit Aβ40 peptide fibrillization and significantly suppress cell cytotoxicity. Formal [4+4] cycloaddition reaction of imines is thus involved in modulating oxidative stress processes associated with neural diseases.

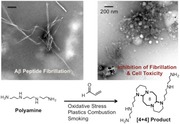

1

Polyamines, such as putrescine, spermidine, and spermine, are polycationic amines that interact with negatively charged molecules (e.g., DNA, RNA, and proteins) to play important roles in a range of biological processes such as cell growth. Present at sub‐millimolar concentrations, their expression levels within mammalian cells are known to be tightly regulated by various biosynthetic, degradation, and transport processes.^1^ Recent studies have suggested that polyamines (along with other biomolecules such as threonine and highly unsaturated lipids) are implicated in the amine oxidase‐based production of acrolein, a highly reactive unsaturated aldehyde,^2^, ^3^, ^4^, ^5^ under oxidative stress conditions.

Acrolein, which can also be produced during the burning of organic materials or smoking,^6^, ^7^ has been shown in literature to react with thiol, hydroxyl, or amino functional groups of DNA, proteins, or phosphatidyl ethanolamines to accelerate oxidative stress processes associated with various disease states (e.g., cancer,^8^ stroke,^9^, ^10^ arteriosclerosis,^11^ or Alzheimer's disease (AD)^12^). As a consequence, the detection of 3‐formyl‐3,4‐dehydropiperidine (FDP), formed from two molecules of acrolein with the ε‐amino group of lysine, is currently employed as an oxidative stress marker.^13^, ^14^, ^15^ In a similar process, polyamines have also been shown to react with acrolein to produce the corresponding FDP derivatives.^16^

With an increase of cellular polyamine or cytotoxic acrolein levels, research has shown a correlation with the progression of certain diseases, such as cancer or stroke.^17^, ^18^ In the brains of AD patients, observed levels of acrolein or spermine (**SPM**) are increased,^19^, ^20^ whereas spermidine (**SPD**) or putrescine levels are decreased.^19^ Recent reports also indicate that polyamines can promote amyloid‐β peptide 1–40 (Aβ40) fibrillization, which is implicated in the acceleration of the AD process.^21^

Recently, we discovered that spermine and spermidine smoothly react with acrolein to produce 1,5‐diazacyclooctanes (cyclic spermine and spermidine, **cSPM** and **cSPD**) through a formal [4+4] cycloaddition of the intermediary unsaturated imines (**Scheme** **1**).^22^, ^23^, ^24^, ^25^ We demonstrated that these compounds are produced in much higher amounts and efficiency than the oxidative stress marker, FDP, which thus far has only been detected under standard analytical conditions. This likely suggests that acrolein reacts with polyamines to exclusively produce the eight‐membered heterocycles as initial acrolein‐modified intermediates. Given these results, there is considerable potential for these compounds to be implicated in biological processes that were previously unexplored or overlooked. This can be supported by our recent demonstration that diazaheterocycles produced from polyamines (e.g., **cSPM**) can efficiently neutralize the toxicity of acrolein, and that eight‐membered polymers produced through sequential cycloaddition processes, both within and on the surface of oxidatively stressed cells, are responsible for damaging cellular function.^22^

**Scheme 1:**
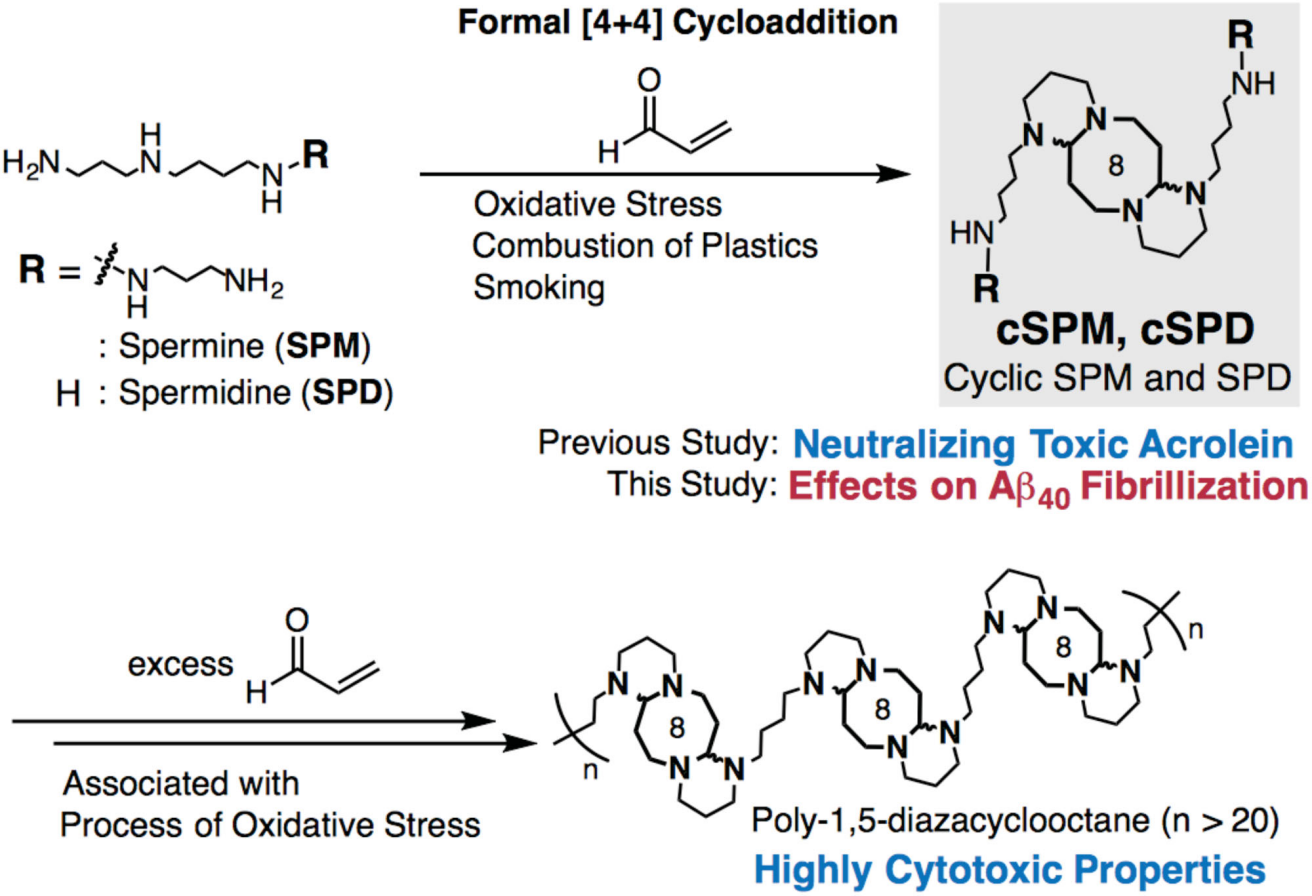
Biological production of 1,5‐diazacyclooctanes through sequential formal [4 + 4] cycloaddition.

2

Our investigations into the biological significance of diazaheterocycles led us to focus on Aβ fibrillization, largely due to the fact that acrolein is produced in the brain tissues of AD patients as a polyamine metabolite during oxidative stress processes. It was speculated that the eight‐membered polyamine–acrolein heterocycles (i.e., **cSPM** or **cSPD**) may potentially control and/or modulate disease progression. Unlike previous reports suggesting that polyamines promote Aβ40 fibrillization,^21^ this study clearly shows that the biologically relevant polyamine–acrolein conjugates inhibit fibrillization and hence cytotoxicity. Thus, the acrolein/polyamine‐derived [4+4] cycloaddition process may effectively modulate the oxidative stress processes associated with neuronal diseases.

We initially investigated the effects of **cSPM** and **cSPD** on Aβ40 fibrillization. Samples were incubated with 25 × 10^−6^ m of the Aβ40 peptide at 37 °C in phosphate buffered saline (PBS) for 5 d, and fibril formation was evaluated based on the thioflavin T (ThT) fluorescence assay (**Figure** **1**). Although **SPM** and acrolein did not show any activity, fibrillization was efficiently inhibited in the presence of **cSPD** and **cSPM** at concentrations exceeding 0.5 × 10^−6^ m. Furthermore, one of the diazacyclooctanes, spermine‐derived **cSPM**, effectively suppressed fibrillization for more than a month (**Figure** **2**), indicating that **cSPM** could steadily inhibit Aβ fibrillization for an extended period of time.

**Figure 1:**
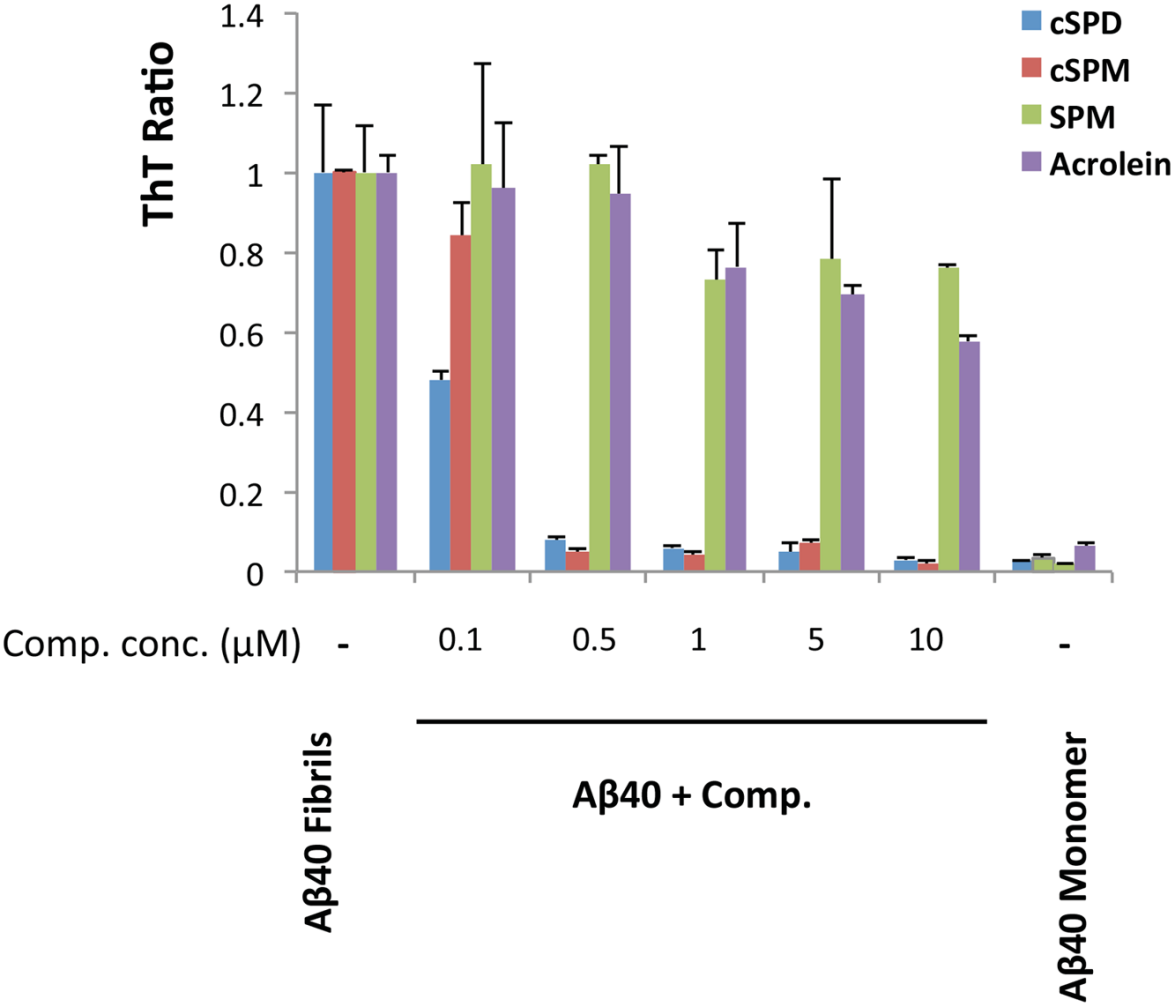
Inhibitory effects of **cSPM** and **cSPD** toward fibrillization of Aβ40. Aβ40 fibrils (control); Aβ was incubated without compounds. Aβ40+comp.; Aβ40 was incubated with compounds (cSPD, cSPM, SPM, or acrolein at 0.1 × 10^−6^, 0.5 × 10^−6^, 1.0 × 10^−6^, 5.0 × 10^−6^, or 10 × 10^−6^ m). Aβ40 monomer (control); Aβ40 was not incubated. Aβ40 peptide (1 × 10^−3^ m stock in DMSO) was diluted to 25 × 10^−6^ m in PBS with our without compounds (stocked in PBS), and incubated at 37 °C for 5 d. For ThT assay, 2.5 μL Aβ samples were mixed with 20 × 10^−6^ m ThT solution in Glycine‐NaOH buffer (50 × 10^−3^ m, pH 8.0), and fluorescent measurements were carried out using plate reader. The average value of three wells was shown. ThT intensity of Aβ40 fibrils was normalized as 100%.

**Figure 2:**
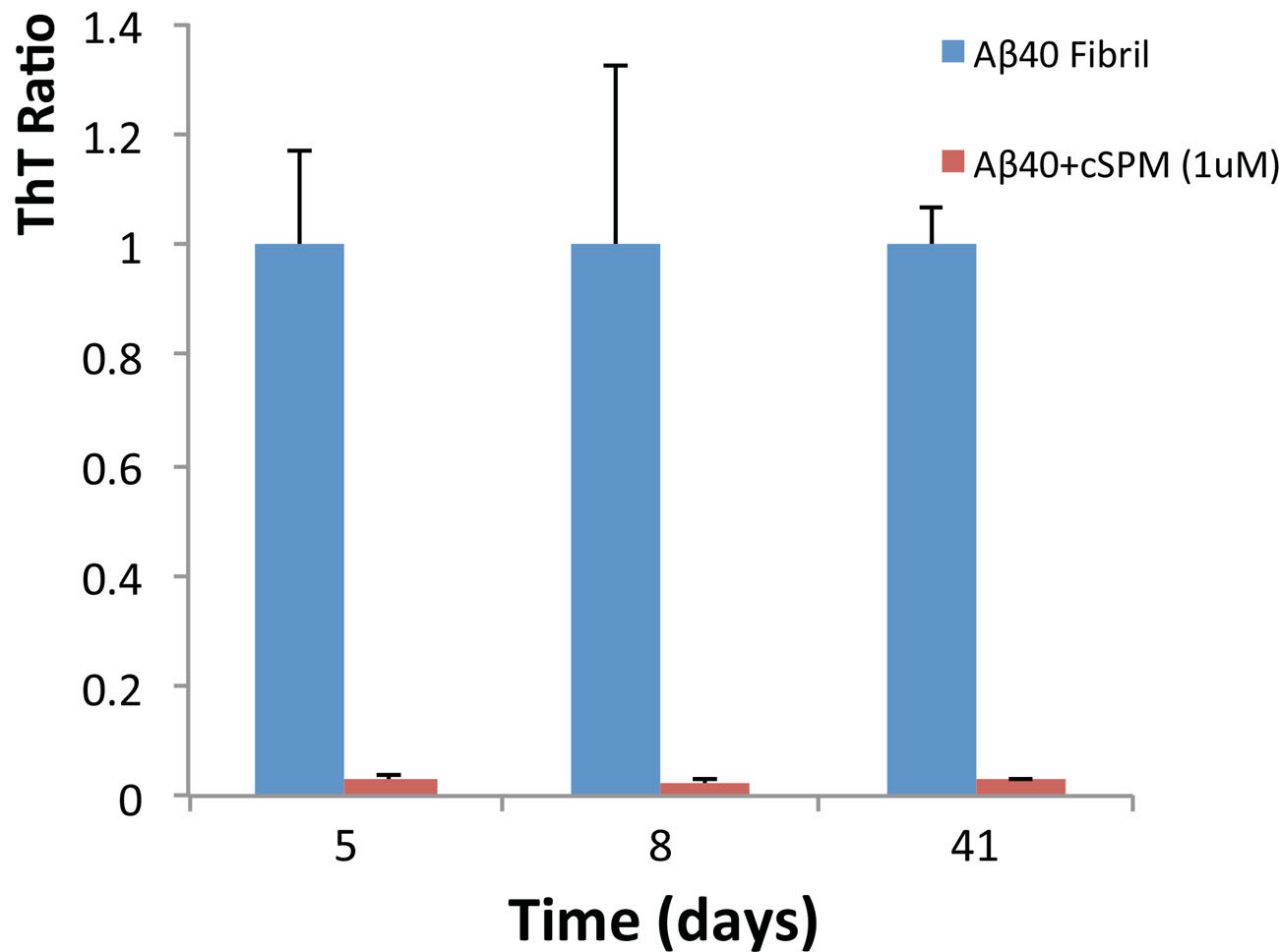
Effect of cSPM on inhibition of Aβ40 fibrillization for an extended period of time. Aβ40 (25 × 10^−6^ m) was incubated with 1 × 10^−6^ m of **cSPM** for indicated time. ThT intensity of Aβ40 fibrils at each incubation time was normalized as 100% (please see the experimental conditions in Figure 1 caption and the Experimental Section).

We next examined the activity of Aβ40 samples treated with **cSPM** using PC12 cells derived from transplantable rat pheochromocytoma (**Figure** **3**). In agreement with previous reports,^25^, ^26^, ^27^ Aβ40 fibrils are expected to display cytotoxic activity. However, observations indicate that with **cSPM** treatment in a dose‐dependent manner, Aβ40 cytotoxicity was notably reduced, along with higher cell viability. It should be further noted that this effect was only seen with **cSPM** concentrations at or greater than 0.5 × 10^−6^ m. At **cSPM** concentrations of 0.1 × 10^−6^ m, cytotoxicity was not observed, which is consolidated by the ThT assay results showing fibrillization could not be inhibited in the presence of 0.1 × 10^−6^ m cSPM (Figure 1).

**Figure 3:**
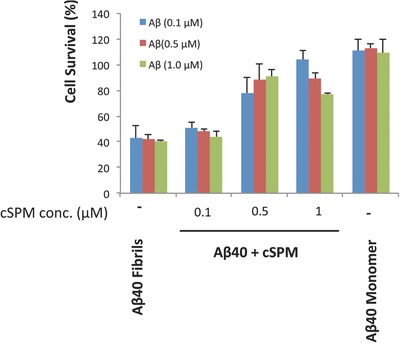
Cytotoxicity of the Aβ40 peptides pretreated with **cSPM** (0.1 × 10^−6^, 0.5 × 10^−6^, or 1.0 × 10^−6^ m) under the conditions described in Figure 1 (37 °C, 5 d). PC12 cells (40 000 cells per well) were incubated in the presence of 0.1 × 10^−6^, 0.5 × 10^−6^, or 1.0 × 10^−6^ m **cSPM**‐treated Aβ40 for 24 h, and cell survival was evaluated using the MTT assay. Monomeric Aβ40 as a negative control was used immediately after dissolving in PBS.

The mechanism by which polyamine–acrolein heterocycle **cSPM** inhibited Aβ40 fibrillization and cytotoxicity was examined by analyzing the molecular sizes of the **cSPM‐**treated Aβ40 peptides using native PAGE/western blotting techniques and an anti‐Aβ40 antibody (6E10) (**Figure** **4**a, also see Figure S3a, Supporting Information). This method permitted detection of the soluble monomer at the bottom of the gel (lane 1), whereas the insoluble aggregates, such as the Aβ40 fibrils, remained at the top (lane 5). Intriguingly, a significant quantity of the Aβ40 monomer remained in the Aβ40 samples after treatment with 0.5 × 10^−6^ or 1.0 × 10^−6^ m **cSPM** (lanes 3 and 4), although some insoluble species were also detected. The “soluble” oligomeric species, which are considered to be highly toxic to cells,^28^, ^29^, ^30^ were not observed in these gels. The absence of notorious “soluble” aggregates, therefore, supported the significant inhibitory effects of **cSPM** on cytotoxicity, as observed in Figure 3.

**Figure 4:**
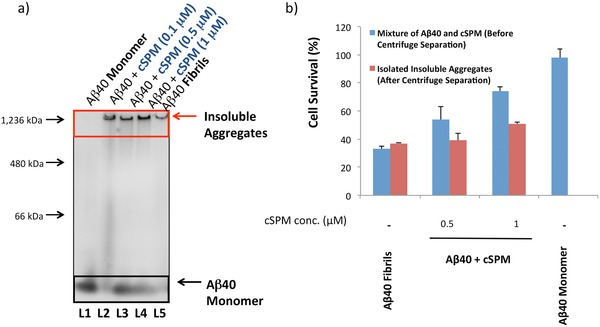
a) Native PAGE/western blots of the **cSPM**‐treated Aβ40 peptides. Lane 1: Aβ40 monomer (control), lanes 2–4: Aβ40 was incubated with 0.1 × 10^−6^, 0.5 × 10^−6^, or 1.0 × 10^−6^ m **cSPM**, respectively, lane 5: Aβ40 fibrils (control). The native marker (Invitorgen) comprising IgM hexamer (1236 kDa), apoferritin (480 kDa), and BSA (66 kDa), which was run separately, was shown as molecular weight marker. b) Comparison of the cytotoxicities of the **cSPM**‐treated Aβ40 mixtures (blue) or insoluble aggregates isolated by centrifugation (red). In assay, PC12 cells (40 000 cells per well) were incubated in the presence of 0.5 × 10^−6^ m cSPM‐treated Aβ40 for over night, and cell survival was evaluated using the MTT assay. The quantity of the insoluble aggregates was calculated by subtracting the amount of the soluble monomeric peptide (estimated by Micro BCA protein Assay Kit (Thermo Fisher Scientific K. K., Waltham, MA, USA)) from total amount of the Aβ40 peptide used in the experiment.

The quantities of the monomeric Aβ40 species present in the **cSPM‐**treated samples in Figure 4a were determined by separating the “insoluble” Aβ40 aggregates from the monomeric peptide by centrifugation (see details in Figure S3a, Supporting Information). The percentages of Aβ40 monomers that remained in the **cSPM‐**treated Aβ40 samples were calculated to be 70%–80% of the mixtures (for samples treated with 0.5 or 1.0 × 10^−6^ m **cSPM**, Figure S3b, Supporting Information). Therefore, it can be concluded that **cSPM** efficiently suppresses insoluble aggregate formation. As a side note, the insoluble species isolated from the **cSPM‐**treated Aβ40 mixture as the minor product was found to match the cytotoxicity of Aβ40 fibrils (prepared as a control) to PC12 cells (Figure 4b). Together with the TEM images of **Figure** **5**, which show that **cSPM** could noticeably reduce fibril formation, the collective data suggest that **cSPM** inhibits cytotoxicity by a mechanism that involves both blocking the formation of highly toxic “soluble” oligomer species and minimizing the formation of toxic “insoluble” Aβ40 fibrils. As a consequence, Aβ40 peptides are expected to be maintained in a monomeric state, thereby reducing cytotoxicity.

**Figure 5:**
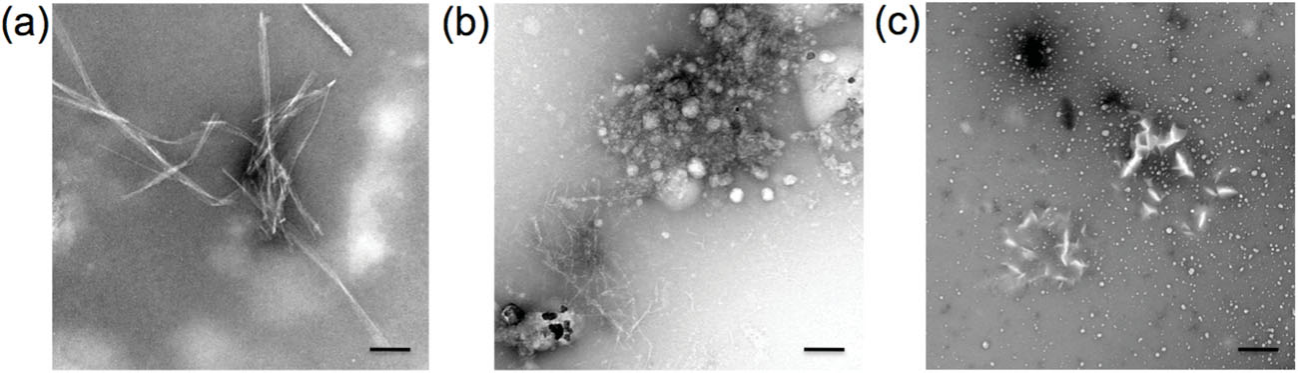
TEM images of Aβ40 fibrillization a) in the absence, or b) in the presence of **cSPM**. c) TEM image of **cSPM** as the control. Scale bars represent 200 nm.

Observations also show that **cSPM** pre‐incubated in PBS solution for various time intervals did not induce decomposition or affect the inhibitory activity (see Figure S4, Supporting Information). These eight‐membered heterocycles also did not produce any conjugate products involving the lysine groups of model peptides. With these points in mind, it can be strongly speculated that **cSPM** was therefore the active structure that inhibited fibril formation.

In addition, NMR studies of the Aβ40 peptide titrated using various concentrations of **cSPM** in PBS revealed chemical shifts at several residues were changed, which includes positions Arg5, His6, Ala21, Ser26, and Asn27 (**Figure** **6** **;** Figure S5, Supporting Information).^31^ Therefore, it could be suggested that these residues may be responsible for suppressing peptide aggregation.

**Figure 6:**
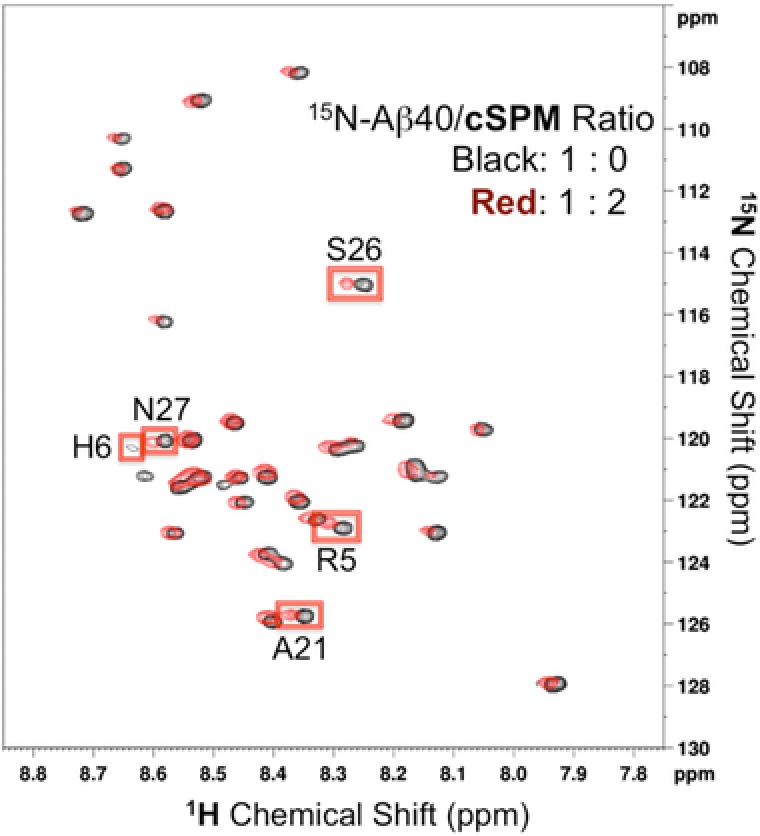
Interaction of Aβ with cSPM. ^5^N‐labeled Aβ40 peptide (0.1 × 10^−3^ m) was mixed with 0.2 × 10^−3^ m cSPM in 50 × 10^−3^ m PBS (pH 6.5, 10% D_2_O) at 5 °C. ^1^H–^15^N HSQC spectra of the ^15^N‐labeled Aβ40 in the absence (black) or presence (red) of cSPM were shown. The red square indicates amino acid residues that showed spectral shift due to possible binding of cSPM with these residues.

Finally, Aβ40 fibrillization was found to be directly suppressed by in situ generated **cSPM** (i.e., by simultaneously treating with polyamine and acrolein) by virtue of the facile [4+4] cycloaddition process that occurs in aqueous media (**Figure** **7**). Note that neither polyamine nor acrolein alone inhibited fibrillization, as shown in Figure 1. Given that these heterocycles were produced in vivo as the oxidative metabolites of the polyamines, a unique strategy for neuronal disease treatment may be envisioned.

**Figure 7:**
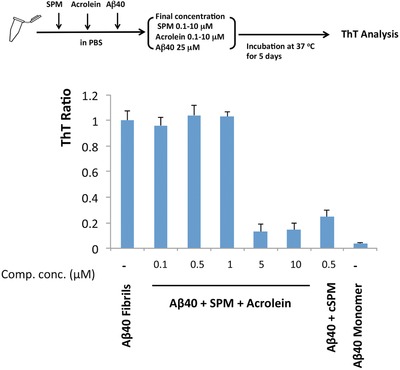
Inhibition of Aβ40 fibrillization by the in situ‐prepared **cSPM**. Aβ40 fibrils (control); Aβ40 was incubated without compounds. Aβ40 + SPM + Acrolein; Aβ40 was incubated with mixture of equimolar SPM and acrolein at 0.1 × 10^−6^, 0.5 × 10^−6^, 1.0 × 10^−6^, 5.0 × 10^−6^, or 10 × 10^−6^ m. Aβ40 + cSPM; Aβ40 was incubated with cSPM (0.5 × 10^−6^ m, control). Aβ40 monomer (control); Aβ40 was not incubated.

In summary, we found that 1,5‐diazacyclooctanes, the exclusive and biologically relevant products between polyamines and acrolein, inhibited Aβ40 peptide fibrillization and significantly suppressed cytotoxicity. These compounds may inhibit the formation of the highly toxic “soluble” oligomer species while minimizing the toxic “insoluble” Aβ40 fibrillization process. There was no significant difference in inhibitory activity between cSPM and cSPD in Figure 1. The results therefore show that the cyclic 1,5‐diazacyclooctane structure is critical to show the activity. The polyamines, SPM and SPD, have different expression level in AD process, but once smoothly reacted with acrolein in/out of the cells under the oxidatively stressed conditions, the corresponding cSPM and cSPD products might similarly inhibit the fibrillization. The results described in this Communication corroborate our discovery that the formal [4+4] cycloaddition reaction is involved in modulating oxidative stress processes associated with neural diseases.

## 3 Experimental Section

*Inhibition of Amyloid Peptide Aggregation*: A stock solution of Aβ40 (1 × 10^−3^ m, solubilized in dimethyl sulfoxide (DMSO)) was diluted to 25 × 10^−6^ m in PBS (pH 7.4) in the presence of various concentrations of **cSPD** and **cSPM** (solubilized in PBS). The DMSO concentration in the solution was kept at a minimum of 2.5% in all experiments. The solution was incubated for 5 d at 37 °C.

*Thioflavin T (ThT) Fluorescence Assay*: The amyloid solution prepared above was diluted to 12.5 × 10^−6^ m in PBS, and 40 μL of the solution was mixed with 160 μL of 25 × 10^−6^ m ThT solution in 50 × 10^−3^ m Glyine‐NaOH buffer (pH 8.0) (final concentration, 2.5 × 10^−6^ m Aβ and 20 × 10^−6^ m ThT). ThT fluorescence was monitored using a spectrofluorometer (Ex = 440 nm, Em = 495 nm). The average values of three‐wells are shown. Experimental results for optimization of the ThT assay conditions are shown in Figure S1a–c (Supporting Information). It was also confirmed that the presence of cSPM does not affect ThT intensity of Aβ fibrils (Figure S1d, Supporting Information). In addition, the effect of DMSO on Aβ40 aggregation was examined and it was shown that although at higher DMSO concentration (10%) Aβ aggregation was almost completely inhibited, significant amount of Aβ aggregation was still observed in the presence of 2.5% DMSO, supporting that cSPM could inhibit Aβ aggregation even in the presence of a small amount of DMSO (Figure S2, Supporting Information).

*Cytotoxicity Assay*: Cell viability was determined using cell proliferation kit (Roche, Basel, Switzerland). PC12 cells (a clonal line of rat pheochromocytoma) were plated in PDL‐coated 96‐well plates at a density of 40 000 cells per well and grown overnight. The **cSPM**‐treated Aβ samples prepared above was diluted with PBS to the various concentrations (20 μL), and were added to the PC12 cells (in 80 μL of medium). The fluorescence intensity of formazan product at 550 nm was measured by microplate reader (see details in the Supporting Information).

## Supporting information

As a service to our authors and readers, this journal provides supporting information supplied by the authors. Such materials are peer reviewed and may be re‐organized for online delivery, but are not copy‐edited or typeset. Technical support issues arising from supporting information (other than missing files) should be addressed to the authors.

Supplementary
