# Supplementary material for: 1,5‐Diazacyclooctanes, as Exclusive Oxidative Polyamine Metabolites, Inhibit Amyloid‐β(1‐40) Fibrillization
Source: Adv Sci (Weinh). 2016 Jun 1;3(10):1600082. doi: 10.1002/advs.201600082 (PMC5096251; doi:10.1002/advs.201600082)
Supplement: Supplementary file 1 — Supplementary [file ADVS-3-0m-s001.pdf]

Copyright WILEY-VCH Verlag GmbH & Co. KGaA, 69469 Weinheim, Germany, 2016.

ADVANCED SCIENCE
Open Access

Supporting Information

for *Adv. Sci.*, DOI: 10.1002/advs.201600082

1,5-Diazacyclooctanes, as Exclusive Oxidative Polyamine Metabolites, Inhibit Amyloid-*β*(1-40) Fibrillization

*Ayumi Tsutsui, Tamotsu Zako, Tong Bu, Yoshiki Yamaguchi, Mizuo Maeda, and Katsunori Tanaka**

Copyright WILEY-VCH Verlag GmbH & Co. KGaA, 69469 Weinheim, Germany, 2013.

Supporting Information

1,5-Diazacyclooctanes, as Exclusive Oxidative Polyamine Metabolites, Inhibit Amyloid-β(1-40) Fibrillization

*Ayumi Tsutsui, Tamotsu Zako, Tong Bu, Yoshiki Yamaguchi, Mizuo Maeda, and Katsunori Tanaka**

Materials. Lyophilized human amyloid-β 1-40 (Aβ40) was obtained from Peptide Institute Inc. (Osaka, Japan). The purity of the sample was certificated by the company. Mouse monoclonal Aβ40 antibody (6E10) was purchased from Abcam (Cambrige, UK). Horseradish peroxidase-conjugated anti-mouse IgG was purchased from R&D systems (Minnespolis, MN, USA). Phosphate Buffered Saline (PBS) was purchased from Life technologies Co. (Carlsbad, CA, USA). Tween-20 and Thioflavin T (ThT) were purchased from Wako Pure Chemical Industries Ltd. (Tokyo, Japan). Tris-Glycine-SDS buffer (TBST) was purchased from Bio-Rad (Hercules, CA, USA). Dimethyl sulfoxide (DMSO) was purchased from Kanto chemical CO., INC. (Tokyo, Japan). Spermine (**SPM**) and spermidine (**SPD**) were purchased from Sigma Aldrich Co. (St. Louis, MO, USA). Cyclic spermine and spermidine, **cSPD** and **cSPM**, were prepared as described previously.[1,2]

Inhibition of amyloid peptide aggregation. A stock solution of Aβ40 (1 mM, solubilized in DMSO) was diluted to 25 µM in PBS (pH 7.4) in the presence of various concentrations of **cSPD** and **cSPM** (solubilized in PBS). The solution was incubated for 5 days at 37 °C.

Thioflavin T (ThT) fluorescence assay. ThT fluorescence assay was performed based on the Lesley's protocol.[3] The amyloid solution prepared above was diluted to 12.5 µM in PBS, and 40 µL of the solution was mixed with 160 µL of 25 µM ThT solution in 50 mM Glyine-

NaOH buffer (pH 8.0) (final concentrations, 2.5 μM Aβ and 20 μM ThT). After 5 min, ThT fluorescence was monitored (Ex =440 nm, Em=495 nm) using a spectrofluorometer (FP-6500 with microplate reader FMP-963; JASCO. Ltd., Tokyo, Japan) equipped with a 96 well plate. The average values of three wells were shown.

For optimization of the assay condition, ThT fluorescence of Aβ fibrils (2.5 μM) at various ThT concentrations (1, 5, 10, 20, 50, 100 and 200 μM) (**Figure S1a**), and ThT fluorescence (20 μM) of Aβ fibrils at various concentrations (0.1, 0.25, 0.5, 1, 2 and 2.5 μM) (**Figure S1b**) were examined. In order to estimate the time dependence, ThT fluorescence intensities at 0, 5, 10 and 20 min after mixing 2.5 μM Aβ and 20 μM ThT were also measured (Figure S1c). Effect of cSPM on ThT intensity was estimated by measuring ThT intensity of Aβ fibrils (2.5 μM) in the absence or presence of cSPM (0.5 and 1.0 μM) (**Figure S1d**). ThT intensity of Aβ fibrils did not change after addition of cSPM. These results indicate that decreased ThT intensities by the compounds are due to inhibition of Aβ aggregation.

Throughout this research, Aβ peptide was initially dissolved in DMSO to prepare a stock solution. The incubation with cSPM, cSPD, SPM and acrolein were performed in PBS, and the DMSO concentration was kept at minimum of 2.5% in all experiments. In order to estimate the effect of DMSO, Aβ40 peptide was incubated in the absence or presence of cSPM (0.5 or 1 μM) in PBS including various concentrations of DMSO (2.5, 5.0 and 10.0%). ThT fluorescence was measured as described above. The amount of Aβ aggregation reduced in a dose dependent manner of DMSO concentration (**Figure S2**). Although at higher DMSO concentration (10%) Aβ aggregation was almost completely inhibited, significant amount of Aβ aggregation was still observed in the presence of 2.5% DMSO. Therefore our results still support that cSPM could inhibit Aβ aggregation even in the presence of a small amount of DMSO.

Cell toxicity assay. Cell viability was determined using cell proliferation kit (Roche, Basel, Switzerland), which is based on the conversion of the tetrazolium salt by mitochondrial dehydrogenase to a formazan product that can be measured spectrophotometrically at 550 nm as described. PC12 cells (a clonal line of rat pheochromocytoma, American Type Culture Collection, Manassas, VA, USA) were maintained in RPMI1640 medium with 10% house serum, 5% fetal bovine serum, 0.1% penicillin, and 0.1% streptomycine in 5% CO_2 at 37 ºC. The cells were plated in PDL-coated 96-well plates at a density of 40,000 cells per well and grown overnight. The **cSPM**-treated Aβ samples prepared above was diluted with PBS to the various concentrations (20 µL), and were added to the PC12 cells (in 80 µL of medium). The fluorescence intensity of formazan product at 550 nm was measured by microplate reader (Tecan, Männedorf, Switzerland). The fluorescence intensity derived from the cells exposed to PBS as the control was used as the 100% viability.

Transmission electron microscope (TEM). TEM images of Aβ aggregates were obtained by using a transmission microscope (JEM-1400, JEOL, Tokyo, Japan) with an acceleration voltage of 80 kV. The Aβ peptide were incubated for 5 days at 37 ºC in the presence of absence of **cSPM**. Approximately, 2 µL of the stained solution by a 2.5% (w/v) samarium acetate solution (2 µL), was placed on 150-mesh copper grid covered with a carbon-coated hydrophilic film. The solution on the grid was allowed and dried before the measurement.

Native PAGE and Western blotting. The **cSPM**-treated Aβ samples were diluted with native page sample buffer (2:1 (v/v), Bio-rad, Hercules, CA, USA), applied to a 5-20% gradient Tris-Glycine precast gel (Wako, Osaka, Japan), run at current of 10 mA, and transferred to a nitrocellulose membrane (0.22 µm) (GE Healthcare Life science, Germany) at current of 100 V for 70 min. The membrane was blocked at room temperature for 1 h with 5% skim milk in TBST including 0.005% tween-20, probed with the mouse monoclonal anti-Aβ 1-16 (6E10, 1:2000) for 1 h at room temperature, followed by washing with TBST and

probing with secondly antibody (HRP-conjugated anti-mouse IgG, 1:1000). Aβ40/antibodies complexes were visualized using the ECL prime blotting detection reagent (GE Healthcare, Little Chalfont, U.K.) according to the manufacture's instructions. Luminescence was detected in a LAS 4000 mini Luminescent Image Analyzer (Fujifilm, Tokyo, Japan), with the Image Reader Las 4000 software.

Separation and quantitative analysis of insoluble Aβ40 aggregates and monomeric peptide: Native PAGE/western blot and dot blot analysis. The **cSPM**-treated Aβ40 samples (total volume of 360 μL) were prepared according to the procedure above. The 260 μL of sample solution was centrifuged by 15,000 rcf for 10 min, and separated into top phase (200 μL), middle phase (55 μL), and bottom phase (5 μL). The success of the separation was confirmed by native PAGE/western blotting analysis of the top and bottom phase samples (Figure S3a). The quantities of monomeric Aβ40 peptide in top and middle phases were then evaluated by using Micro BCA™ protein Assay Kit (Thermo Fisher Scientific K. K., Waltham, MA, USA). Insoluble aggregates being present at bottom phase was estimated by subtracting the amount of monomeric peptide (i.e,. in top and middle phases) from total amount of the Aβ40 peptide used for the experiment (***Note: staining intensity on the gel in Figures 4a and S3a do not correlate with real quantities of the peptide***). Cytotoxicity of the mixture of Aβ40 and **cSPM** (before centrifuge separation) and insoluble aggregates (after centrifuge separation) was examined by MTT method (**Figure 4b**).

Furthermore, the quantities of monomeric Aβ40 peptide was also evaluated by dot blot analysis (Figure S3b). Two μL of the top phase of centrifuged samples and the mixtures of Aβ40 and **cSPM** (before centrifuge separation) were blotted on nitrocellulose membrane for 5 times. After blocked with 5% skim milk in TBST including 0.005% tween-20 for 1 h at room temperature, the membrane was probed initially with the mouse monoclonal anti-Aβ 1-16

(6E10), and then HRP-conjugated anti-mouse IgG as the secondary antibody, as described previously. After treating with the ECL prime blotting detection reagent, luminescence was detected in a LAS4000 mini Luminescent Image Analyzer. The dot intensity was evaluated by *Image J* software (NIH-USA). The dot intensity of the remained Aβ sample in the supernatant after the centrifugation (Aβ monomer) was compared with the dot intensity of the samples before centrifugation (normalized as 100%). Aβ40 monomeric peptide and fibrils were used as controls.

NMR studies of Aβ40 peptide titrated with cSPM. ^{15}N-labeled Aβ(1-40) (^{15}N-Aβ40) and deuterium oxide (D, 99.9%) were obtained from COSMO Bio Co Ltd and Wako Pure Chemical Industries Ltd (Tokyo, Japan), respectively. ^{1}H-^{15}N HSQC spectra of ^{15}N-Aβ40 were measured on Bruker Biospin Avance 600 spectrometer employing TOPSPIN 2.1 application with a TXI probe. The probe temperature was set to 5 ^{o}C. 1.0 mg ^{15}N-Aβ40 was dissolved at a concentration 114 µM in 50 mM PBS (10% D_2O, pH 6.5). **cSPM** was dissolved at a concentration 20 mM PBS (10% D_2O, pH 6.5) and added to the ^{15}N-Aβ40 solution. The final concentration of ^{15}N-Aβ40 and **cSPM** was 100 µM and 200 µM, respectively. The pH of the resulting ^{15}N-Aβ40 mixture was slightly shifted to 6.53 by adding the **cSPM** solution. **cSPM** inhibited the Aβ40 fibrillization at 200 µM at this pH range. ^{1}H NMR chemical shifts indicated with parts per million (ppm) were calibrated based on an outer standard of a chemical shift of 4,4-dimethyl-4-silapentane-1-sulfonic acid (DSS), given a singlet at 0 ppm. ^{15}N chemical shifts (ppm) are calibrated using indirect reference based on the IUPAC-IUB recommended ^{15}N/^{1}H resonance ratio of 0.10132911.[4] ^{1}H-^{15}N HSQC spectra assignment of ^{15}N-Aβ40 was conformed by Hoshino's report.[5] Changes in chemical shifts were calculated by following equation;[6]

$$\Delta\delta = [(\Delta\delta_H)^2 + (0.2 \text{ X } \Delta\delta_N)^2]^{1/2}$$

where $\Delta\delta_H$ and $\Delta\delta_N$ are the observed chemical shift change for ^{1}H and ^{15}N, respectively.

Reference

[1] A. Tsustui, R. Imamaki, S. Kitazume, S. Hanashima, Y. Yamaguchi, M. Kaneda, S. Oishi, N. Fujii, A. Kurbangaliva, N. Taniguchi, K. Tanaka, *Org. Biomol. Chem.* **2014**, *12*, 5151.

[2] A. R. Pradipta, A. Tsutsui, A. Ogura, S. Hanashima, Y. Yamaguchi, A. Kurbangaliva, K. Tanaka, *Synlett* **2014**, *25*, 2442.

[3] K. M. Sörgjerd, T. Zako, M. Sakono, P. C. Stirling, M. R. Leroux, T. Saito, P. Nilsson, M. Sekimoto, T. C. Saido, M. Maeda, *Biochemistry* **2013**, *52*, 3532.

[4] D. S. Wishart, C. G. Bigam, J. Yao, F. Ablidgaard, H. J. Dyson, E. Oldfield, J. L. Markley, B. D. Sykes, *J. Biomol. NMR.* **1995**, *6*, 135.

[5] T. Yamaguchi, K. Matsuzaki, M. Hoshino, *FEBS Lett.* **2011**, *585*, 1097.

[6] M. Kanagawa, Y. Liu, S. Hanashima, A. Ikeda, W. Chai, Y. Nakano, K. Kojima-Akikawa, T. Feizi, Y. Yamaguchi, *J. Biol. Chem.* **2014**, *289*, 16954.

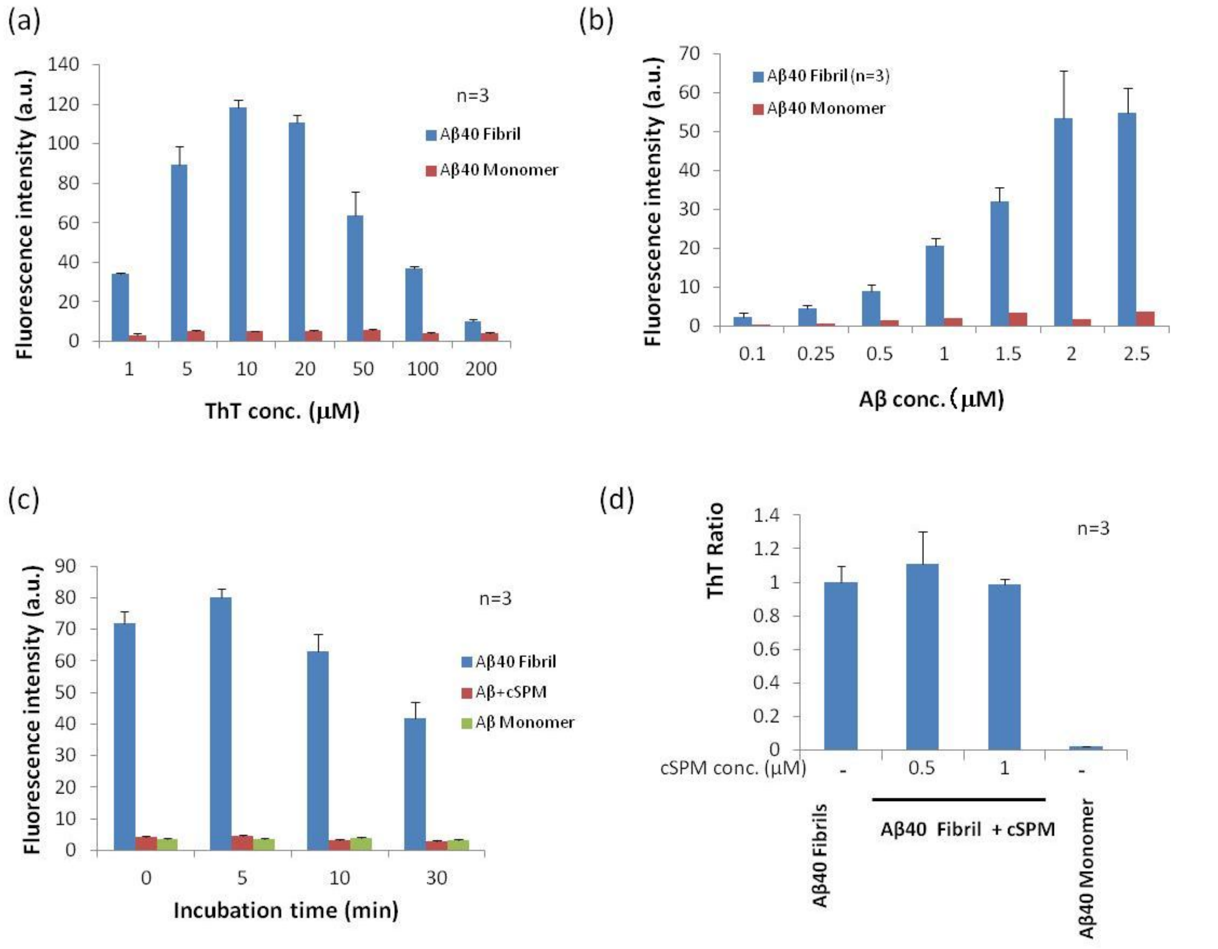

Figure S1. (a) ThT fluorescence of Aβ fibrils (2.5 μM) at various ThT concentrations (1, 5, 10, 20, 50, 100 and 200 μM). (b) ThT fluorescence (20 μM) of Aβ fibrils at various concentrations (0.1, 0.25, 0.5, 1, 2 and 2.5 μM). (c) ThT fluorescence intensities at 0, 5, 10 and 20 min after mixing 2.5 μM Aβ and 20 μM ThT. (d) Effect of cSPM on ThT intensity. ThT intensity of Aβ fibrils (2.5 μM) in the absence or presence of cSPM (0.5 and 1.0 μM) was measured.

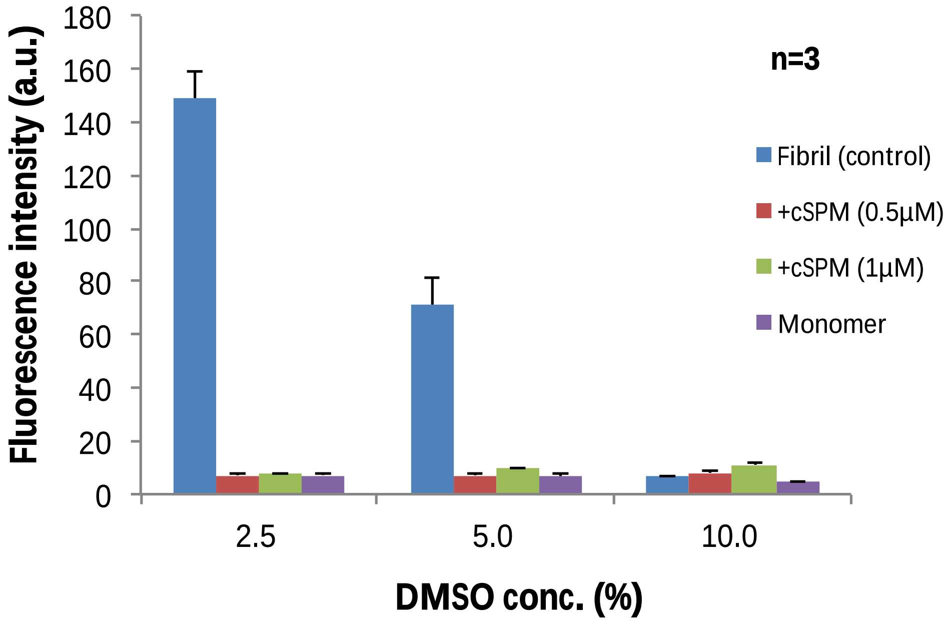

Figure S2. Effect of DMSO. Aβ40 peptide was incubated in the absence or presence of cSPM (0.5 and 1 µM) in PBS including various concentrations of DMSO (2.5, 5.0 and 10.0%). ThT fluorescence was measured as described above.

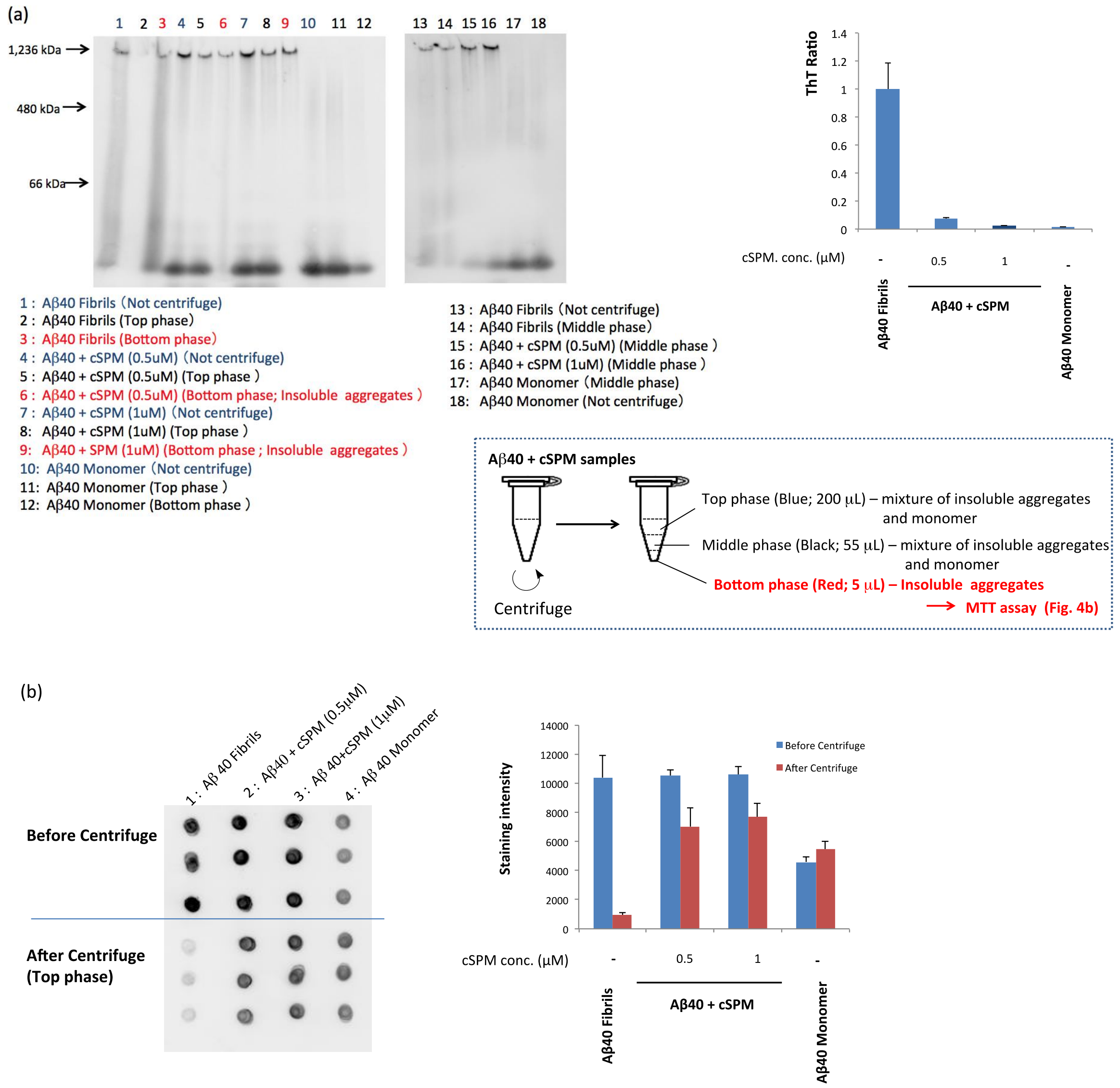

Figure S3. (a) Native PAGE/western blots of the **cSPM**-treated Aβ40 peptide, before and after centrifuge separation. The native marker (Invitorgen) comprising IgM hexamer (1,236 kDa), apoferritin (480 kDa) and BSA (66 kDa), which was run separately, was shown as molecular weight marker. Samples marked by blue (before centrifuge) and red (after centrifuge; bottom phase) labels were subjected to the cytotoxicity assay in Figure 4b. ThT intensity of the samples (blue, before centrifuge) was also shown (right). (b) Dot blot analysis. Dot intensity was evaluated by *Image J* software (NIH, USA).

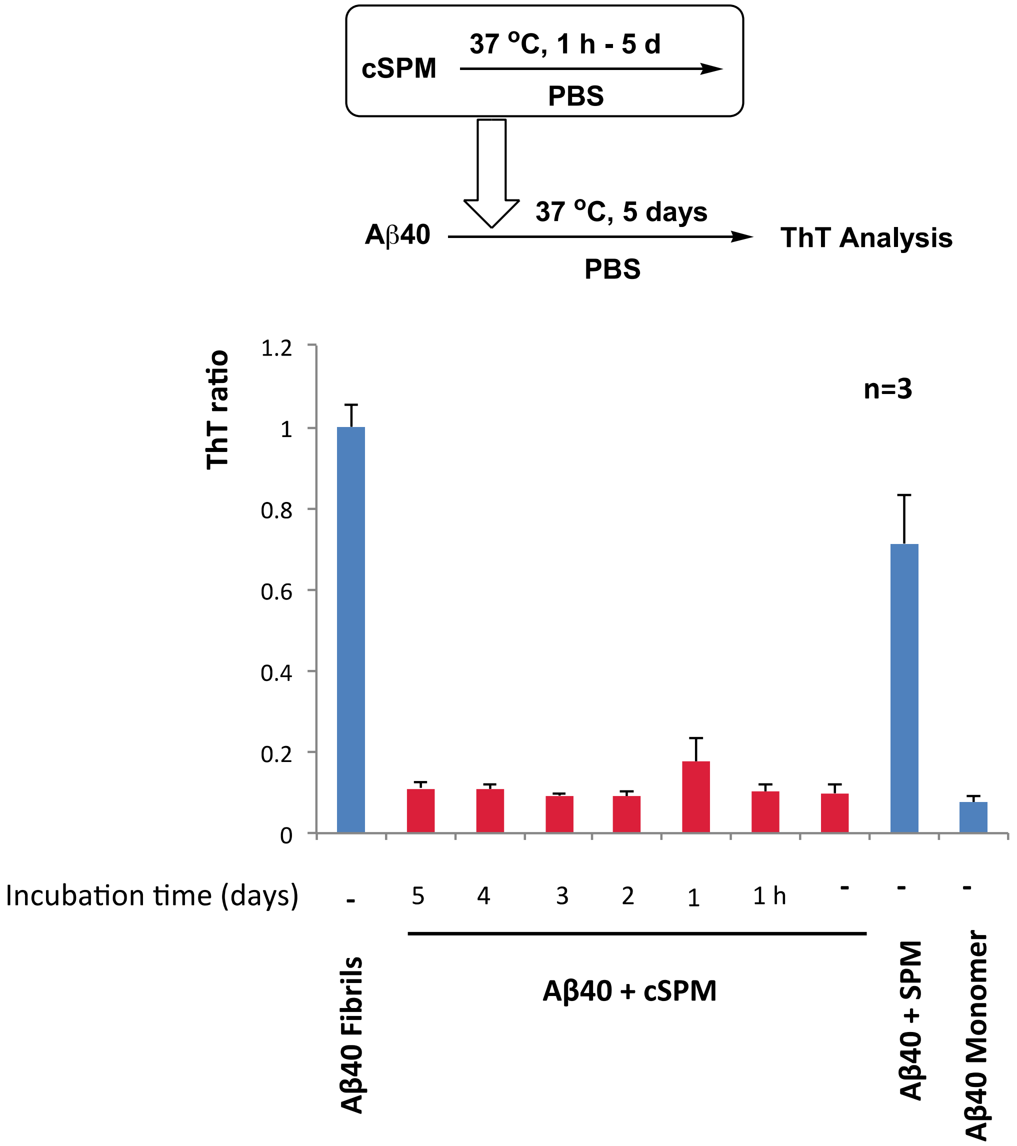

Figure S4. Inhibition of Aβ40 fibrillization by **cSPM** pre-incubated in a PBS for various time intervals (fibrillization properties were evaluated by ThT method as described previously).

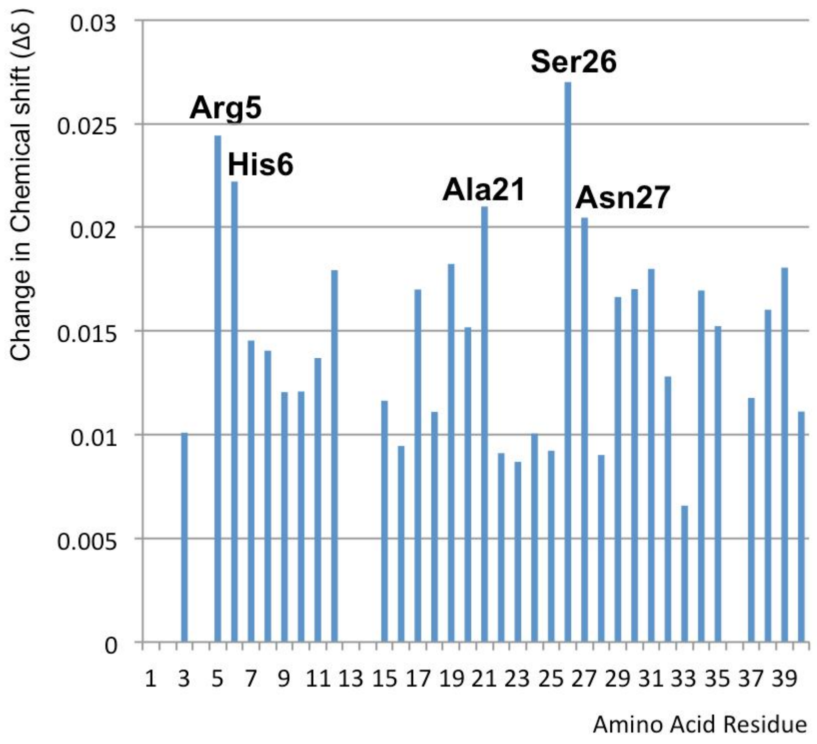

Figure S5. Changes in chemical shifts (Δδ) calculated based on ^{1}H-^{15}N HSQC spectra of Aβ40 peptide titrated with **cSPM** (Aβ40 peptide/cSPM ratio from 1:0 to 1:2). Notably changes of more than Δδ =0.02 could be observed for the residues at Arg5, His6, Ala21, Ser26, and Asn27.
